# 

**Published:** 1885-09

**Authors:** 


PAGE.

ORIGINAL COMMUNICATIONS.

A Method of Detecting Lacerations of the Cervix Uteri Post Partum. John Bartlett ........ 209
Oleate of Manganese. F. H. Martin ........ 211
Does Tobacco Impair Sight? W. F. Coleman ........ 216

EDITORIAL.

The International Medical Congress of 1887 ........ 228
Cholera ........ 229
W. Lawson Tait on The Modern Treatment of Uterine Myoma ........ 231
Dr. Ferran and Cholera—Inoculation ........ 232

CORRESPONDENCE.

Bryonia Alba. C. S. Lombard ........ 234

SOCIETY PROCEEDINGS.

*Chicago Medical, Meeting August 3d.* Oleate of Manganese. F. H. Martin ........ 237
Does Tobacco Impair Sight? W. F. Coleman ........ 238
Laceration of the Cervix Uteri. J. Bartlett ........ 240
*Chicago Medical Society, August 17th.* A Case of Ovariotomy. C. J. Parkes ........ 242
A Case of Battey's Operation. C. J. Parkes ........ 244
A Case of Double Ovariotomy. C. J. Parkes ........ 245
Observations on the Cause and Treatment of Infantile Eczema and Allied Eruptions. H. T. Byford ........ 249
Annual Report of the Department of Health of Chicago, for 1884 ........ 253

BOOK REVIEWS ........ 284

ABSTRACTS AND EXTRACTS.

Progress of Pharmacology and Therapeutics ........ 291
Digestive Ferments ........ 295
Verdict of Court-Martial ........ 297
The Toxicity of Normal Urine ........ 298
The Treatment of Bromidrosis of the Feet ........ 300

BOOKS RECEIVED ........ 289

PAMPHLETS RECEIVED ........ 290

NEWS ITEMS ........ 303

LOCAL ITEMS ........ 304

